# Patient-aware single-cell analysis of fibrovascular remodeling in a predominantly colorectal cancer immunotherapy cohort

**DOI:** 10.3389/fimmu.2026.1961075

**Published:** 2026-10-01

**Authors:** Yang Gu, Li Li, Yuxian Zhang, Jie Tang, Zhigang Wang, Xiaofeng Luo

**Affiliations:** 1 Department of Hepatobiliary and Pancreatic Surgery, Ward I, Jingmen Central Hospital, Jingmen Central Hospital affiliated to Jingchu University of Technology, Jingmen, Hubei, China; 2 Operating Room, Jingmen Central Hospital, Jingmen Central Hospital affiliated to Jingchu University of Technology, Jingmen, Hubei, China

**Keywords:** cancer-associated fibroblast, colorectal cancer, immune checkpoint blockade, pericyte, single-cell RNA sequencing

## Abstract

**Background:**

Cancer-associated fibroblasts (CAFs) and vascular mural cells may shape extracellular matrix organization in colorectal cancer. However, single-cell comparisons are susceptible to unequal cell recovery, ambient RNA, doublets, treatment-stage mixing, and repeated sampling within patients. We investigated fibrovascular remodeling using patients as the inferential unit and distinguished reproducible signals from donor-concentrated and context-dependent findings.

**Methods:**

We reanalyzed a published single-cell RNA sequencing cohort of 22 patients, including 20 with colorectal cancer and 2 with duodenal carcinoma. The primary pericyte analysis included 14 pretreatment matched normal-tumor pairs with at least three pericytes per tissue. We assessed ambient contamination and doublets using DecontX and scDblFinder. CAFs were reclustered at six response-blind resolutions, and the stable parent population was tested by patient-blocked pseudobulk analysis after excluding its dominant donor. Independent CRC single-cell and spatial datasets, a cross-cancer immunotherapy cohort, and TCGA were used for external testing.

**Results:**

After DecontX correction, tumor pericytes had higher ECM6 scores (median paired change 0.309; false discovery rate [FDR] 0.018). Scores were also higher for the matrix stiffness transcriptional proxy (0.277; FDR 0.012) and the hypoxia/angiogenesis transcriptional program (0.255; FDR 0.028). All three signatures remained significant at the five-pericyte threshold (n=13). COL1A1 and FN1 increased in the analysis, although COL1A1 was borderline in the five-cell sensitivity analysis. Response-blind stability analysis identified a 2,983-cell ECM-remodeling CAF population represented in all 22 patients. After excluding P02, patient-blocked pseudobulk analysis in 15 patients showed higher matrix, inflammatory, and myCAF-related programs and a lower iCAF program. Neither SPP1 nor baseline enrichment in stable disease was supported. In GSE205506, a disjoint 10-gene fibroblast gate retained higher inflammatory and composite CAF scores in non-pCR samples. The highest-scoring unsupervised cluster was donor-concentrated and showed no patient-level association with response. Spatial and cross-cancer analyses yielded mixed or null results.

**Conclusions:**

These results support pretreatment fibrovascular transcriptional remodeling at the patient level. They do not establish an SPP1-high response biomarker, a mechanical stiffness phenotype, or causal CAF-to-pericyte signaling. Donor-aware analysis places the rare-cluster observation within a broader, reproducible ECM-remodeling CAF program while defining its evidential limits.

## Introduction

Immune checkpoint blockade has transformed treatment for colorectal cancers with mismatch repair deficiency or microsatellite instability (1–3). However, clinical benefit remains heterogeneous. Recent CRC studies implicate stromal architecture and extracellular matrix organization in variation in immune access and treatment response (4–6). Spatial and single-cell analyses further show that tumor, stromal, and immune compartments form clinically relevant niches (7–9).

Cancer-associated fibroblasts (CAFs) organize the tumor microenvironment by remodeling collagen, fibronectin, and basement membrane components and producing inflammatory mediators (10–12). They can also suppress effector T-cell activity (13). Recent spatial studies link fibroblast-rich structural boundaries and invasive-front programs to restricted immune access or local tumor progression (14–16). Other studies have identified distinct stromal programs at the CRC leading edge (17). Pericytes form the vascular mural compartment and may influence endothelial stability, basement membrane composition, and phenotypic plasticity in CRC (18–20). Yet, the patient-level relationship among CAF states, pericyte remodeling, and treatment heterogeneity remains unresolved.

SPP1 encodes a secreted phosphoprotein involved in adhesion, inflammatory signaling, and matrix remodeling. However, SPP1 is not CAF-specific and is expressed by myeloid and other stromal populations in CRC (10, 21). Its interpretation therefore depends on the cellular state in which it is expressed; SPP1 alone cannot define the cell of origin or treatment response. This distinction is critical in droplet-based single-cell data. Unequal cell recovery, ambient RNA, doublets, treatment-stage mixing, and repeated sampling can otherwise create precise-looking but donor-driven associations.

Here, we used a patient-aware framework to address three linked objectives. First, we assessed whether pretreatment tumor pericytes showed matched transcriptional remodeling in extracellular matrix, matrix stiffness-related and hypoxia/angiogenesis-related programs after contamination, and doublet sensitivity analyses. Second, we determined whether the rare CAF_C16 split lay within a stable cross-patient CAF population rather than a response-associated state. Finally, we tested whether independent CRC datasets supported a composite fibroblast program, SPP1 alone, or neither. Positive, mixed, and negative external results were retained to define the limits of the proposed fibrovascular model.

## Materials and methods

### Study design, source data, and cohort metadata

The discovery data were generated in a published longitudinal single-cell study of immunotherapy-treated gastrointestinal tumors and are available under GEO accession GSE236581 (6). We analyzed the processed CAF and pericyte objects together with the official patient and sample metadata. The cohort comprised 20 patients with CRC and 2 with duodenal carcinoma. Cancer type, stage, mismatch repair (MMR) status, microsatellite instability (MSI) status, treatment regimen, response, and sampling stage were transcribed from the original **Supplementary Table 1**. P01 was recorded as dMMR/MSS and P16 as pMMR/MSI in that source table. Because assay-level records were not available in the public files, MMR and MSI were retained as separate source variables and were not recoded or harmonized.

The response field in the project object was reconciled patient by patient against the official source table before analysis. P03 and P05 were corrected from complete response (CR) to partial response (PR), and P20 and P22 were corrected from PR to CR. Normal tissue was not treated as a response group. Discovery-cohort response strata are reported as CR, PR, and stable disease (SD); the terms sensitive and insensitive were not used as clinical classifications. External cohorts retained their original endpoints, including pathological complete response (pCR) and non-pCR.

### Single-cell objects, sampling counts, and primary pericyte inclusion

The complete compartment-specific objects contained 21,349 CAFs and 1,578 pericytes across normal and tumor tissues and all available treatment stages. The full GSE236581 metadata contained 975,275 quality-controlled cells. For each patient and tissue, CAF and pericyte abundance was therefore also calculated as cells per 1,000 quality-controlled cells using the full-cell denominator. Absolute compartment counts and CAF:pericyte composition are retained as sampling audits. Cell-level UMAPs and pooled cell counts are descriptive only (**Supplementary Figure S3**; **Supplementary Table S8**).

The primary pericyte analysis was restricted to Treatment I, defined in the source metadata as pretreatment baseline. Patients were included when both matched normal and tumor tissue contained at least three pericytes, yielding 14 paired patients. Sensitivity analyses used a stricter threshold of at least five pericytes in each tissue (n=13) and a broader threshold of at least one pericyte in each tissue (n=17). Later treatment stages were excluded from the primary paired analysis. The patient, rather than the individual cell, was the inferential unit.

### Ambient-RNA, doublet, and quality-control analyses

The source objects contained filtered count matrices, but unfiltered empty droplets were unavailable. Consequently, SoupX contamination fractions could not be estimated (22). We combined baseline CAF and pericyte raw counts in a joint mesenchymal SingleCellExperiment. DecontX 1.18.2 then estimated cell-level contamination and generated corrected counts without requiring an empty-droplet matrix (23). Raw-count analyses were retained as sensitivity analyses.

scDblFinder 1.16.0 was applied to the joint baseline mesenchymal object (24). Several patient-tissue strata contained too few pericytes for stable independent doublet models. We therefore performed joint classification and treated the results as a technical audit. Primary analyses retained all DecontX-corrected cells, whereas sensitivity analyses excluded predicted doublets. **Supplementary Figure S1** and **Supplementary Table S2** report cell counts, detected genes, contamination estimates, doublet scores, raw-versus-corrected ECM6 concordance, and patient-tissue pericyte counts. Because tissue dissociation can alter stromal recovery and induce stress programs, we treated these effects as unresolved technical limitations (25, 26).

### Normalization and transcriptional program scores

Raw and DecontX-corrected counts were normalized to counts per 10,000 transcripts and transformed using log1p. Each score was the arithmetic mean of the available normalized genes. Seurat AddModuleScore and UCell were not used. **Supplementary Table S2** provides the complete gene sets, overlap audits, and calculation formulas.

ECM6 comprised COL4A1, COL4A2, LAMA4, LAMA2, NID1, and HSPG2. The matrix stiffness transcriptional proxy comprised LOX, LOXL1, LOXL2, LOXL3, LOXL4, PLOD1, PLOD2, PLOD3, P4HA1, P4HA2, P4HA3, TGM2, COL1A1, COL1A2, COL3A1, COL5A1, COL5A2, COL11A1, COL12A1, CTGF, CYR61, ANKRD1, and AMOTL2. These genes capture collagen production, maturation, crosslinking, and matrix-responsive transcription implicated experimentally in tissue stiffening (20); the score is not a mechanical measurement or a validated stiffness classifier. The hypoxia/angiogenesis transcriptional program comprised MMP2, MMP14, VEGFA, ANGPT2, SERPINE1, THBS1, TIMP1, HIF1A, PDGFB, and CXCL12.

The fibroblast non-ECM control comprised PRRX1, TWIST2, and DCN, and the pericyte identity control comprised RGS5, PDGFRB, MCAM, CSPG4, and NOTCH3. To separate the score tests from individually tested sentinel genes, ECM4 retained COL4A2, LAMA2, NID1, and HSPG2, while the stiffness-core score removed COL1A1 and retained the remaining 22 stiffness-proxy genes. COL1A1, COL4A1, FN1, LAMA4, and LUM were tested individually. ECM6 and the full stiffness proxy do not share genes; the disjoint sensitivity removes genes evaluated separately as individual features.

### Patient-level statistical analysis

Cell-level scores were averaged within each patient and tissue. The primary contrast was the paired tumor-minus-normal difference. Two-sided paired Wilcoxon signed-rank tests were used, with Benjamini–Hochberg correction applied separately to three prespecified signatures and five prespecified individual genes. The two disjoint signatures formed a separate correction family. Median paired changes and 95% confidence intervals were estimated from 2,000 patient-level bootstrap resamples.

Prespecified sensitivity analyses used raw counts, predicted singlets, CRC-only patients, disjoint scores, a five-pericyte threshold, and an any-cell threshold. The fibroblast non-ECM and pericyte identity controls comprised one prespecified negative-control family, and their P values were adjusted together. Spearman correlation quantified the association between the baseline tumor CAF:pericyte ratio and the paired ECM6 change. No individual cell was treated as an independent replicate in response-associated inference.

### CAF resolution stability and patient-blocked pseudobulk analysis

The CAF object was processed using Seurat 5.3.0 and SeuratObject 5.1.0 (27, 28). Counts were log-normalized with a scale factor of 10,000, and 3,000 variable features were selected. After scaling and principal component analysis, the nearest-neighbor graph and UMAP embedding were constructed using principal components 1-20. Louvain clustering was repeated at fixed resolutions of 0.05, 0.10, 0.20, 0.30, 0.40, and 0.50. Response labels were not used for clustering or resolution selection. At each resolution, we defined the parent as the cluster containing the largest number of CAF_C16 cells. Resolution selection considered adjacent-resolution membership stability, patient coverage, largest-donor fraction, and P02 fraction. Resolution 0.20 was the highest resolution within the stable 0.05-0.20 plateau.

For differential expression, tumor CAFs from P02 were excluded. Patients were retained if they contributed at least 20 cells to both the ECM-remodeling parent population and the remaining CAF compartment. A total of 15 patients met these criteria. Raw counts were summed by patient and state. edgeR 4.0.16 used TMM normalization, filterByExpr, robust dispersion estimation, and a quasi-likelihood model with patient and state terms. P values were adjusted across genes using the Benjamini–Hochberg method. Prespecified program scores were calculated from pseudobulk log counts per million and compared using paired Wilcoxon tests. P values were adjusted across five programs. Baseline response comparisons used patient-level parent-population fractions and did not inform clustering-resolution selection. This patient-level pseudobulk design reduces false precision from cell-level replication (29, 30).

The original 109-cell CAF_C16 split was retained only for traceability. Because 108 cells came from P02 Treatment II, marker testing was repeated within P02 Treatment II by comparing the 108 rare-split cells with 29 other CAFs. SPP1 detection was defined as normalized expression greater than zero, and Benjamini–Hochberg correction was applied across the marker table. These cell-level results were not used for cohort-level inference.

### Same-patient cell-cell communication analysis

CellChat 2.2.0.9001 was run only on tumor cells from P02 Treatment II (31). The object contained 108 rare-split CAFs, 29 other CAFs, and 11 pericytes. We used the human CellChat database and trimean expression estimator, with a minimum of 10 cells per group. Sender-to-pericyte probabilities were summarized by sender and pathway. Because CellChat combines transcript abundance with ligand-receptor prior knowledge, it ranks transcript-compatible interactions rather than demonstrating ligand binding or causal signaling (31–33).

### Independent colorectal cancer single-cell analyses

GSE178341 was analyzed using the published stromal annotations (34). Composite fibroblast scores and pericyte ECM scores were summarized by patient, and Spearman correlations used the patient as the unit of analysis. Published fibroblast subtype labels were retained to map the composite program to established states; cells were not reselected with the tested ECM score.

For GSE205506, treated tumor samples with pCR or non-pCR metadata were retained (2). The downloadable processed GEO files did not include the published cell-type labels. Fibroblast candidates were therefore selected using a positive lineage score based on PDGFRA, FAP, COL6A1, COL6A2, COL6A3, DPT, C7, CFD, PI16, and PDPN. Immune, epithelial, endothelial, and pericyte lineage scores were required not to exceed the fibroblast score. None of the 10 positive gate genes occurred in the SPP1, ECM, inflammatory, or composite CAF scores. The primary gate threshold was 0.35, with 0.25 and 0.50 used in sensitivity analyses. Continuous metrics were summarized by patient, and P values from two-sided Wilcoxon tests were adjusted across five prespecified metrics.

The disjoint-gated GSE205506 fibroblasts were also analyzed without response labels. After log-normalization and selection of 2,500 variable features, Harmony provided sample-level integration, followed by unsupervised clustering into 12 clusters. The cluster with the highest median composite CAF score was selected before response testing. Patient-level cluster fractions were compared using Wilcoxon tests, and P values were adjusted across all 12 clusters. Cluster size, represented-patient count, and largest-donor fraction were reported.

### Spatial, cross-cancer, and TCGA boundary analyses

The Zenodo 14602110 CRC spatial resource was analyzed using the provided coordinates and labels (35). Neighborhood scores were calculated from the nearest spatial observations within each batch, and labels were permuted within batches. We refer to the observations as spatial units because cell-level resolution was not established for every point.

GSE267401 Visium data were analyzed by sample (36). In-tissue spots were retained, SPP1-high spots were defined within each sample, and ECM and pericyte scores were calculated from available genes. Six-neighbor scores used array coordinates with label permutation within sample. Visium spots are 55-μm multicellular spatial units and were not called cells or used to validate CellChat interactions (37, 38).

IMvigor210 served as a cross-cancer boundary cohort (39). Non-response was defined as SD or progressive disease, whereas response was defined as CR or PR. Composite CAF, pericyte ECM, cytotoxicity, and SPP1 scores were evaluated using exploratory logistic regression, receiver operating characteristic analysis, and survival analysis. The cohort was not treated as CRC validation. TCGA-COAD/READ primary-tumor RNA sequencing data were obtained through the Genomic Data Commons (40, 41). FPKM-UQ values were log2(x+1) transformed and standardized by gene across 637 tumors. P values for seven prespecified bulk-score correlations were adjusted using the Benjamini–Hochberg method. TCGA was used only to assess bulk coexpression.

### Software and reproducibility

Analyses were performed in R version 4.3.3. Primary packages were Seurat 5.3.0, SeuratObject 5.1.0, edgeR 4.0.16, limma 3.58.1, DecontX/celda 1.18.2, and scDblFinder 1.16.0. Additional packages were SingleCellExperiment 1.24.0, CellChat 2.2.0.9001, Harmony 1.2.3, data.table 1.17.4, ggplot2 3.5.2, and patchwork 1.3.0. **Supplementary Tables S1**–**S8** provide the statistical source tables, complete gene-set definitions, and sensitivity analyses.

## Results

### Cohort audit defines the patient-level analytical frame

The cohort comprised 22 patients: 20 with CRC and 2 with duodenal carcinoma (P11 and P24) (**Figure 1A**; **Supplementary Table S1**). The official source table classified 12 patients as CR, 7 as PR, and 3 as SD. A total of 16 tumors were recorded as mismatch repair-deficient and six as mismatch repair-proficient. Similarly, 16 tumors were recorded as microsatellite instability-high and six as microsatellite-stable. MMR and MSI were retained as separate source variables. Treatment was heterogeneous: 12 patients received pembrolizumab, 4 received sintilimab, 1 received camrelizumab, and 5 received checkpoint blockade with CapeOx.

**Figure 1:**
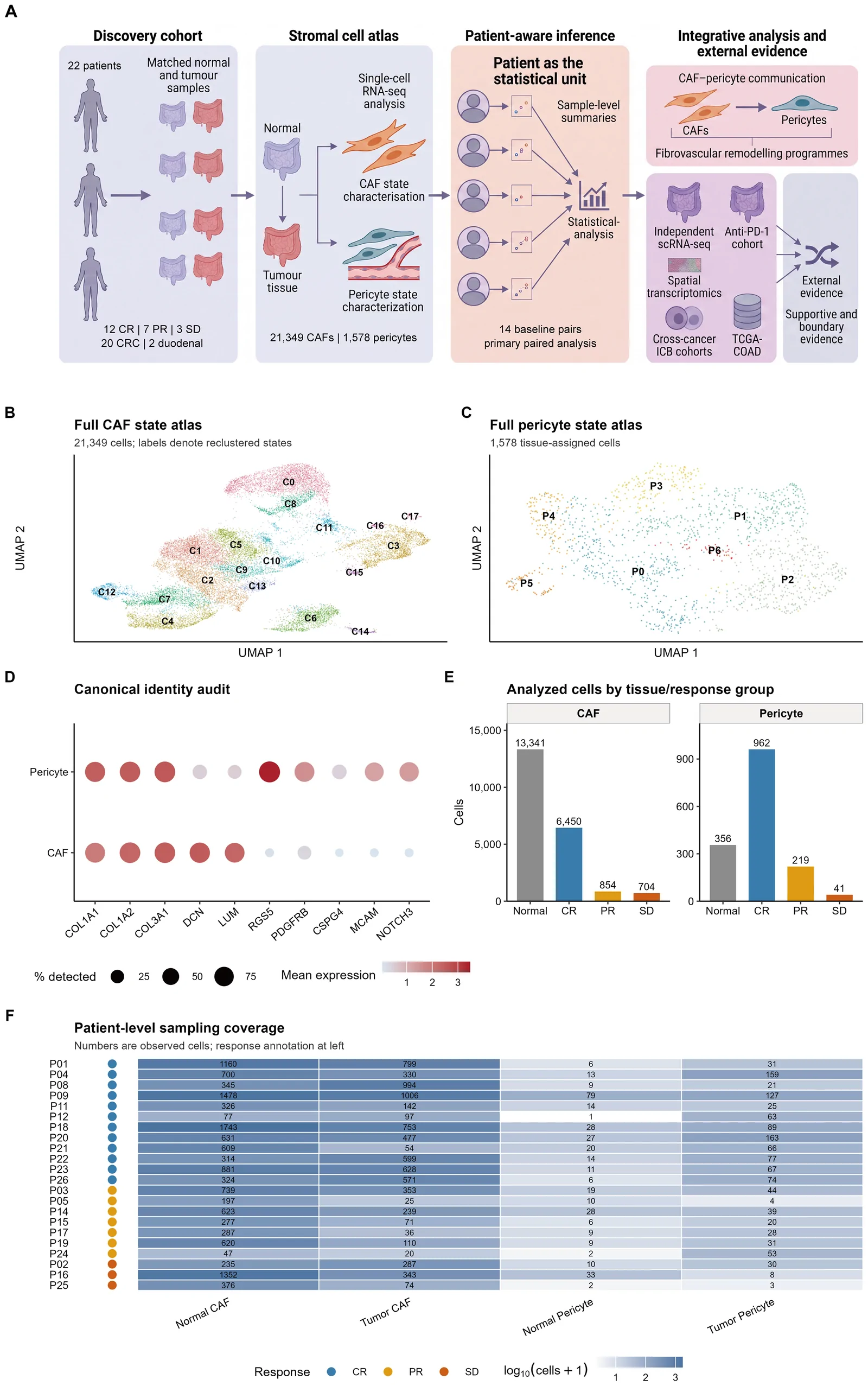
Cohort design, stromal atlas, and sampling structure. **(A)** Study design and cohort composition, including the 14-pair pretreatment primary analysis. **(B)** UMAP of 21,349 CAFs from the full compartment object. **(C)** UMAP of 1,578 pericytes. **(D)** Canonical fibroblast and pericyte identity audit. **(E)** Corrected absolute cell counts in normal, CR, PR, and SD groups. **(F)** Patient-level normal and tumor CAF/pericyte yields and compartment composition. Normalized CAF and pericyte abundances per 1,000 quality-controlled cells are reported in **Supplementary Figure S3**. Cell counts and embeddings are descriptive; inferential analyses use patient-level summaries.

Reconciliation against the official source table corrected four patient-response labels: P03 and P05 from CR to PR, and P20 and P22 from PR to CR. Although the total response-group sizes were unchanged, the correction altered patient-level and cell-level group assignments. All reported analyses use the official response labels (**Supplementary Table S1**).

Across all available stages, the compartment objects contained 21,349 CAFs and 1,578 pericytes. Using the reconciled response labels, pooled CAF counts were 13,341 in normal tissue, 6,450 in CR tumors, 854 in PR tumors, and 704 in SD tumors. The corresponding pericyte counts were 356, 962, 219, and 41 (**Figures 1B–E**). The full metadata contained 975,275 quality-controlled cells. Normalized CAF and pericyte abundances per 1,000 quality-controlled cells varied substantially by patient, tissue, and treatment stage. Consequently, pooled compartment totals were not treated as inferential sample sizes (**Figure 1F**; **Supplementary Figure S3**; **Supplementary Tables S2**, **S8**).

### Pretreatment pericyte remodeling persists across contamination and doublet sensitivity analyses

A total of 14 patients had at least three pretreatment pericytes in both matched normal and tumor tissue: 13 with CRC and 1 with duodenal carcinoma. The primary set included 8 CR, 4 PR, and 2 SD patients. A total of 17 patients contributed to the any-cell sensitivity analysis. Later treatment stages were not pooled with baseline samples.

Median DecontX contamination estimates were 0.072 in CAFs and 0.084 in pericytes. Raw and corrected pericyte ECM6 scores were strongly concordant (Spearman ρ=0.992). Predicted doublets comprised 0.7% of baseline CAFs and 15.6% of baseline pericytes. The comparatively high pericyte doublet fraction motivated a singlet-only sensitivity analysis and limits cell-level interpretation (**Supplementary Figure S1**).

In the primary DecontX analysis, the median tumor-minus-normal change in ECM6 was 0.309 (paired Wilcoxon P = 0.0120; FDR = 0.0181). The median change in the matrix stiffness transcriptional proxy was 0.277 (P = 0.00388; FDR = 0.0116). The hypoxia/angiogenesis transcriptional program increased by a median of 0.255 (P = 0.0280; FDR = 0.0280) (**Figures 2A, B**; **Supplementary Table S3**). COL1A1 increased by a median of 1.344 (FDR = 0.0426), and FN1 increased by 1.457 (FDR = 0.0210). COL4A1, LAMA4, and LUM did not remain significant after correction (FDR = 0.194, 0.754, and 0.227, respectively).

**Figure 2:**
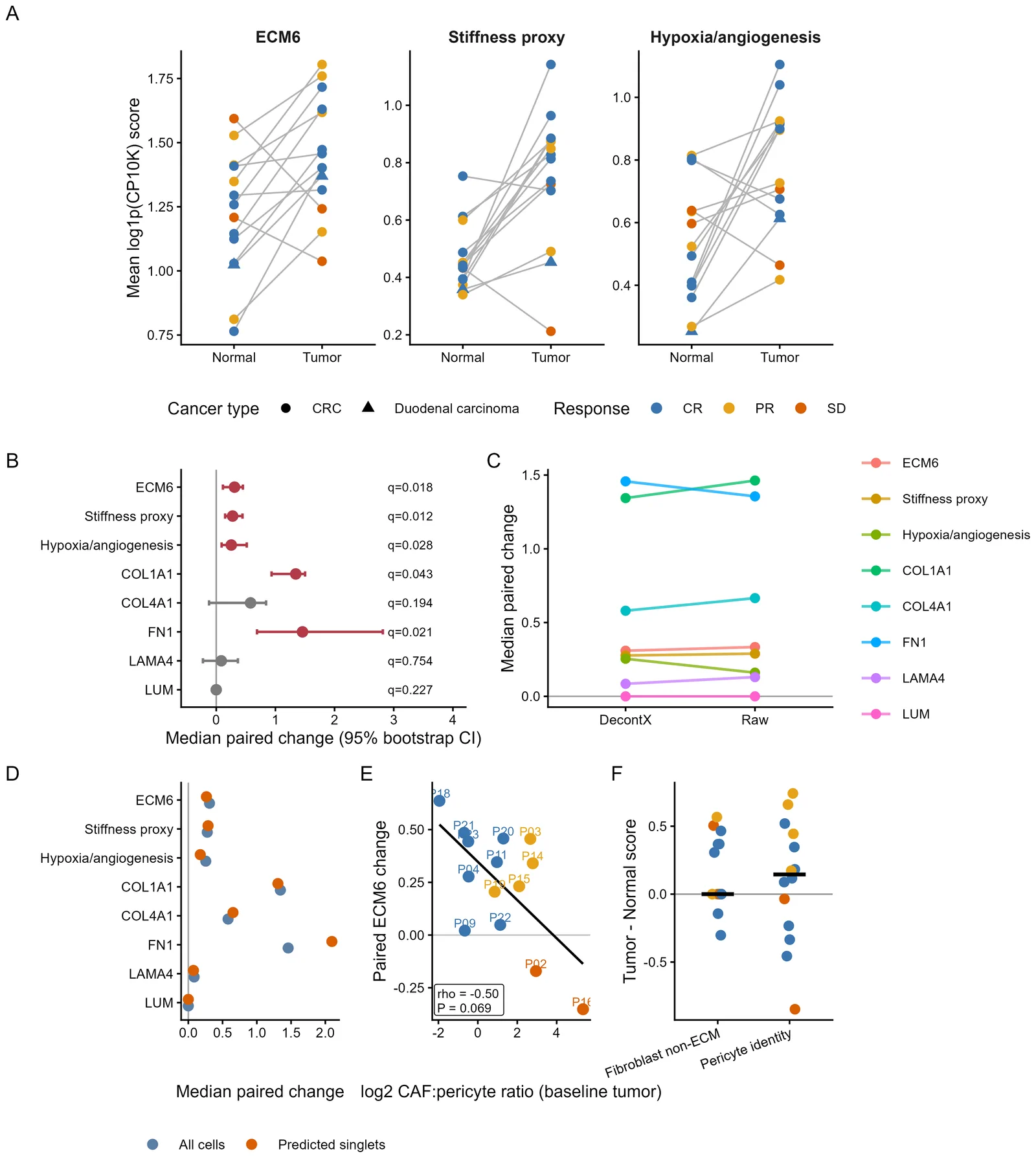
Pretreatment-matched pericyte transcriptional remodeling after ambient-RNA correction. **(A)** Matched normal and tumor ECM6, matrix stiffness transcriptional proxy, and hypoxia/angiogenesis transcriptional program scores in 14 patients with at least three pericytes per tissue. Colors denote official response, and triangles denote duodenal carcinoma. **(B)** Median paired changes and 95% bootstrap confidence intervals with FDR values. **(C)** Raw and DecontX-corrected estimates. **(D)** All-cell and predicted-singlet sensitivity analyses. **(E)** Association between the baseline tumor CAF:pericyte ratio and paired ECM6 change. **(F)** Fibroblast non-ECM and pericyte identity controls. A stricter analysis requiring at least five pericytes per tissue is reported in **Supplementary Table S7**.

Sensitivity analyses yielded consistent effect directions. Raw-count analyses retained higher ECM6, matrix stiffness proxy, hypoxia/angiogenesis transcriptional program, and COL1A1 and FN1 scores. ECM4 and stiffness core remained significant after removing the separately tested sentinel genes (FDR = 0.0202 and 0.00946). After predicted doublets were removed, ECM6, the matrix stiffness proxy, the hypoxia/angiogenesis transcriptional program, COL1A1, and FN1 remained significant (**Figures 2C, D**). Their respective FDR values were 0.0280, 0.0142, 0.0280, 0.0359, and 0.0210. At the five-pericyte threshold (n=13), ECM6, the matrix stiffness proxy, the hypoxia/angiogenesis transcriptional program, FN1, ECM4, and stiffness core remained significant. Their respective FDR values were 0.0262, 0.0193, 0.0303, 0.0335, 0.0360, and 0.0158. COL1A1 was borderline after correction (FDR = 0.0633).

Estimates were less precise in CRC-only analyses. Among all DecontX-corrected cells, ECM6, the matrix stiffness proxy, and FN1 remained significant (FDR = 0.0262, 0.0155, and 0.0335, respectively). The hypoxia/angiogenesis transcriptional program was borderline (FDR = 0.0504). In CRC-only predicted singlets, the matrix stiffness proxy and FN1 remained significant. ECM6 and the hypoxia/angiogenesis transcriptional program each had an FDR of 0.0504, and COL1A1 had an FDR of 0.0527. Thus, effect directions were stable, but several estimates depended on the inclusion threshold and the two non-CRC patients.

The baseline tumor CAF:pericyte ratio was not positively associated with the paired ECM6 change. The DecontX-corrected association was negative but non-significant (ρ=−0.499; P = 0.0694). The raw-count association was negative and nominally significant (ρ=−0.587; P = 0.0274), whereas tumor ECM6 alone was unrelated to the ratio (ρ=−0.204; P = 0.483) (**Figure 2E**). The fibroblast non-ECM control had a negligible median paired change (3.59×10^−10^; P = 0.0371). This result did not remain significant after correction with the pericyte identity control (FDR = 0.0742). The pericyte identity control did not change (median change 0.145; P = 0.391; FDR = 0.391). Neither control was significant at the five-cell threshold (FDR = 0.151 and 0.442) (**Figure 2F**). These controls weigh against a simple positive stromal-loading explanation but do not exclude residual technical variation.

### Resolution stability identifies a cross-patient ECM-remodeling CAF population

Across the six fixed resolutions, the cluster containing CAF_C16 remained stable from 0.05 to 0.20 and contained 2,921, 3,061, and 2,983 cells, respectively. The adjacent-resolution Jaccard index between 0.10 and 0.20 was 0.968. At resolution 0.40, the population separated into the original 109-cell cluster. Resolution 0.20 was selected as the highest resolution within the stable plateau before any response comparison (**Figure 3A**).

**Figure 3:**
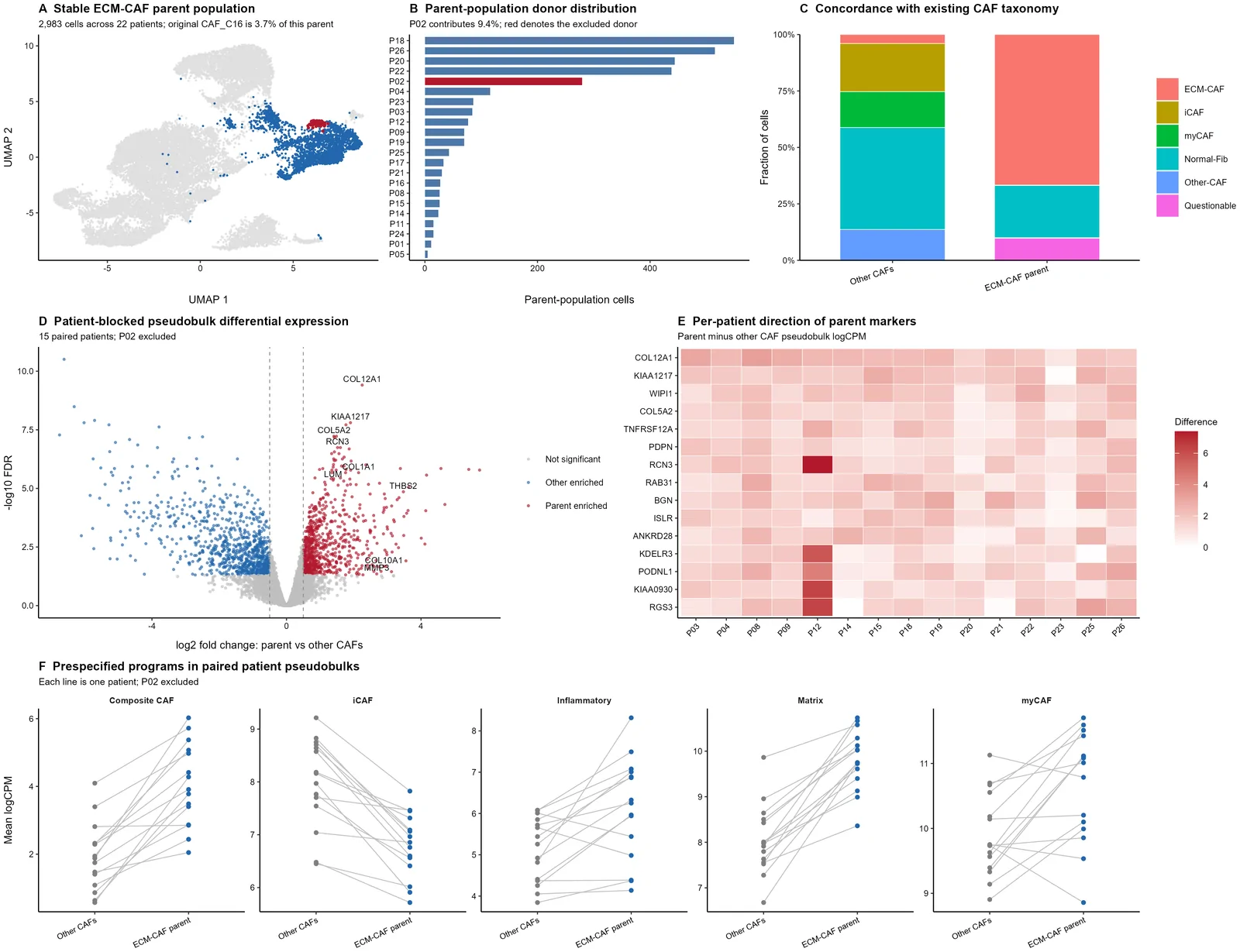
Cross-patient ECM-remodeling CAF population identified by resolution stability and patient-blocked pseudobulk analysis. **(A)** Resolution-0.20 parent population and the nested original CAF_C16 cells in the CAF UMAP. **(B)** Donor distribution; P02 is highlighted and excluded from pseudobulk testing. **(C)** Concordance with the existing CAF taxonomy. **(D)** Patient-blocked edgeR differential expression in 15 paired tumor pseudobulks after excluding P02. **(E)** Per-patient direction of leading parent-enriched genes. **(F)** Paired pseudobulk scores for five prespecified programs. Clustering and resolution selection did not use response labels.

The resolution-0.20 population contained 2,983 cells from all 22 patients, with every patient contributing at least five cells. P18, the largest donor, contributed 18.4%, and P02 contributed 279 cells (9.4%). The population captured all 109 original CAF_C16 cells, which comprised only 3.7% of the parent. Existing annotations classified 66.7% of the population as ECM-CAF, and 98.2% of its cells came from tumor tissue (**Figures 3B, C**). We therefore refer to this resolution-stable parent as a cross-patient ECM-remodeling CAF population.

After excluding P02, 15 patients each contributed at least 20 parent-population CAFs and 20 other tumor CAFs. Patient-blocked edgeR pseudobulk analysis identified 768 parent-enriched genes at FDR<0.05 and absolute log2 fold change >0.5 (**Figures 3D, E**; **Supplementary Table S4**). Matrix, inflammatory, composite CAF, and myCAF-related programs were higher, whereas the iCAF program was lower. All five paired comparisons remained significant after correction (FDR 0.0012-0.0115) (**Figure 3F**). Parent-enriched genes included COL12A1, COL1A1, LUM, THBS2, COL10A1, and MMP3. COL4A1, FN1, and LAMA4 did not remain significant after correction, and SPP1 did not meet pseudobulk expression filtering.

There was no evidence that the parent population was enriched in SD at pretreatment baseline. At resolution 0.20, median patient-level fractions were 0.716 in CR, 0.596 in PR, and 0 in SD (Kruskal–Wallis P = 0.398). The same result was observed across the fixed-resolution series. The population therefore represents a reproducible ECM-remodeling program rather than a response-associated subtype.

### The original CAF_C16 split is a P02-dominated high-resolution subdivision

The 109-cell CAF_C16 split was retained for traceability (**Supplementary Figure S2**). P02 contributed 108 Treatment II cells, whereas corrected-CR patient P20 contributed one Treatment II cell. Within P02 Treatment II, SPP1 was detected in 41 of 108 rare-split cells and 14 of 29 other CAFs (38.0% versus 48.3%). No marker remained significant after multiple-testing correction. Same-patient CellChat retained collagen-, laminin-, and FN1-compatible pathways but not an SPP1 pathway. These findings provide no evidence of cohort-level SD enrichment, an SPP1-defined state, or causal CAF-to-pericyte communication.

### Independent CRC datasets favor a composite fibroblast program over SPP1 alone

In the treatment-naive GSE178341 atlas, the discovery-derived composite CAF score mapped most strongly to published GREM1-positive and MMP3-positive CAF populations (**Figures 4E, F**). Among 59 eligible patients, the composite score correlated with pericyte ECM at the patient level (ρ=0.506; P = 4.40×10^−5^; FDR = 1.32×10^−4^) (**Figures 5A–C**). SPP1 detection alone was not significantly correlated with pericyte ECM (ρ=0.215; FDR = 0.154). This analysis maps the program to established fibroblast states but does not establish a new CAF taxonomy.

**Figure 4:**
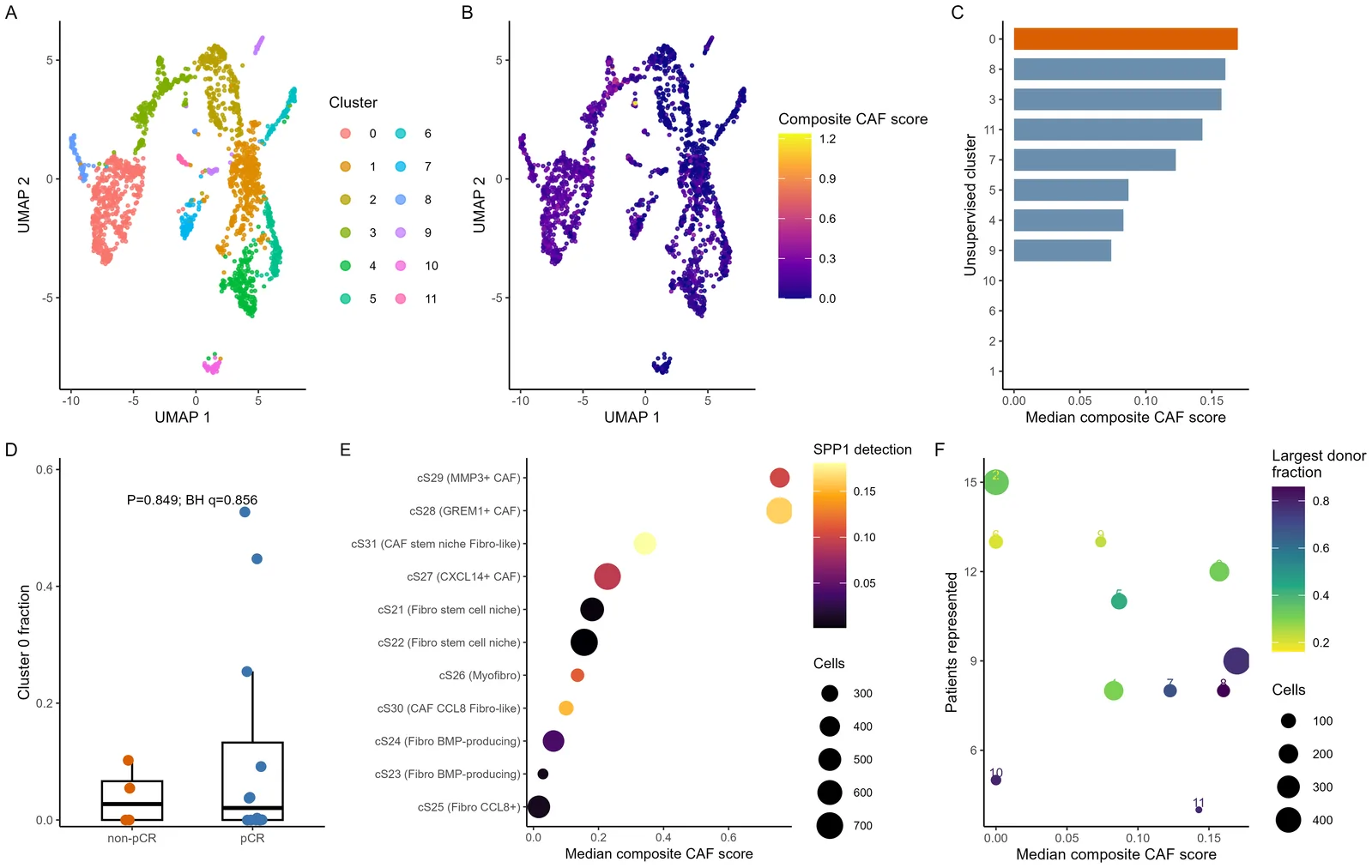
Unsupervised external fibroblast-state analysis and mapping to published CAF states. **(A)** Response-blind clusters among disjoint-gated GSE205506 fibroblast candidates. **(B)** Composite CAF score across the UMAP. **(C)** Median composite CAF score by cluster. **(D)** Patient-level fraction of the highest-scoring cluster 0 in pCR and non-pCR samples; P = 0.849 and BH FDR = 0.856 across all 12 clusters. **(E)** Composite CAF score and SPP1 detection in published GSE178341 fibroblast states. **(F)** Represented-patient count and largest-donor fraction for GSE205506 clusters. Cluster 0 contained 471 cells from 9 patients, but its largest-donor fraction was 76.6%. It was therefore considered donor-concentrated and did not support a discrete response-associated state.

**Figure 5:**
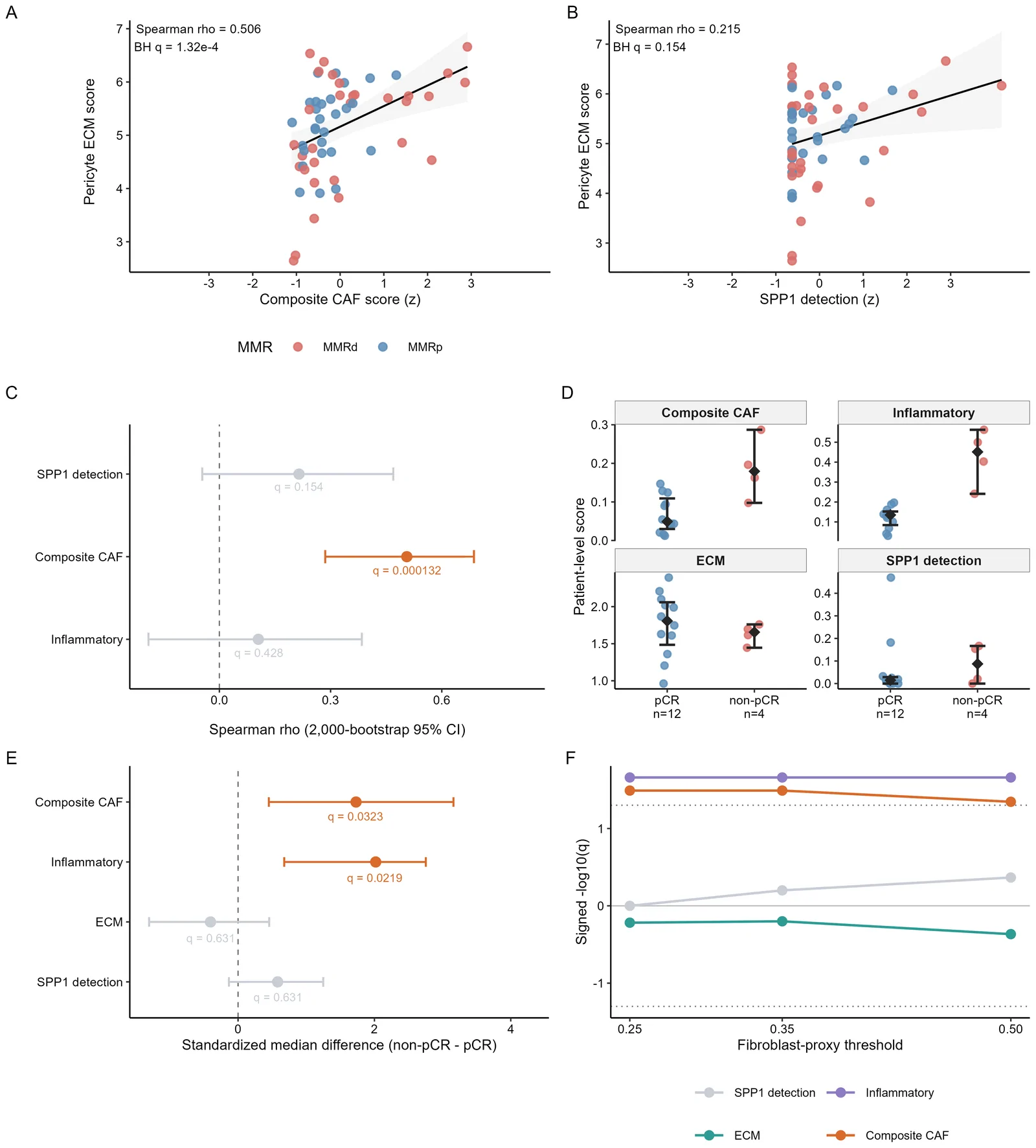
Continuous external colorectal cancer single-cell analyses. **(A–C)** Patient-level association between the composite CAF program and pericyte ECM in treatment-naive GSE178341. **(D–F)** Patient-level inflammatory, composite CAF, ECM, and SPP1 metrics in the GSE205506 anti-PD-1 cohort after a disjoint fibroblast lineage gate. Median inflammatory scores were 0.451 in non-pCR samples and 0.135 in pCR samples. Corresponding composite CAF scores were 0.179 and 0.0491. SPP1 and ECM metrics were not significant after correction. Panel **(F)** shows threshold sensitivity for the disjoint gate.

GSE205506 included 12 pCR and 4 non-pCR patients. With the disjoint fibroblast lineage gate, inflammatory scores were higher in non-pCR samples (median 0.451 versus 0.135; Wilcoxon P = 0.00437; FDR = 0.0219). Composite CAF scores were also higher in non-pCR samples (0.179 versus 0.0491; P = 0.0129; FDR = 0.0323). SPP1 detection, SPP1 expression, and ECM scores did not differ after correction. The inflammatory and composite CAF results remained significant at gate thresholds of 0.25, 0.35, and 0.50 (**Figures 5D–F**; **Supplementary Table S7**).

Response-blind clustering of the disjoint-gated GSE205506 fibroblasts identified 12 clusters. The cluster with the highest composite score contained 471 cells from 9 patients, but one donor contributed 76.6% of its cells (**Figures 4A–F**). Its median patient-level fraction was 0.0273 in non-pCR patients and 0.0205 in pCR patients (P = 0.849; FDR = 0.856 across 12 clusters) (**Figure 4D**). This donor-concentrated cluster showed no evidence of a reproducible response-associated CAF state.

### Spatial and cross-cancer analyses define negative and context-dependent boundaries

In the Zenodo 14602110 spatial resource, SPP1-high stromal neighborhoods had lower pericyte-like and ECM proxy scores than comparison neighborhoods. The resource lacked a dedicated pericyte annotation and did not establish cellular resolution for every spatial observation. We therefore interpreted this result as a negative spatial test rather than evidence of cell–cell communication (**Figures 6A–C**).

**Figure 6:**
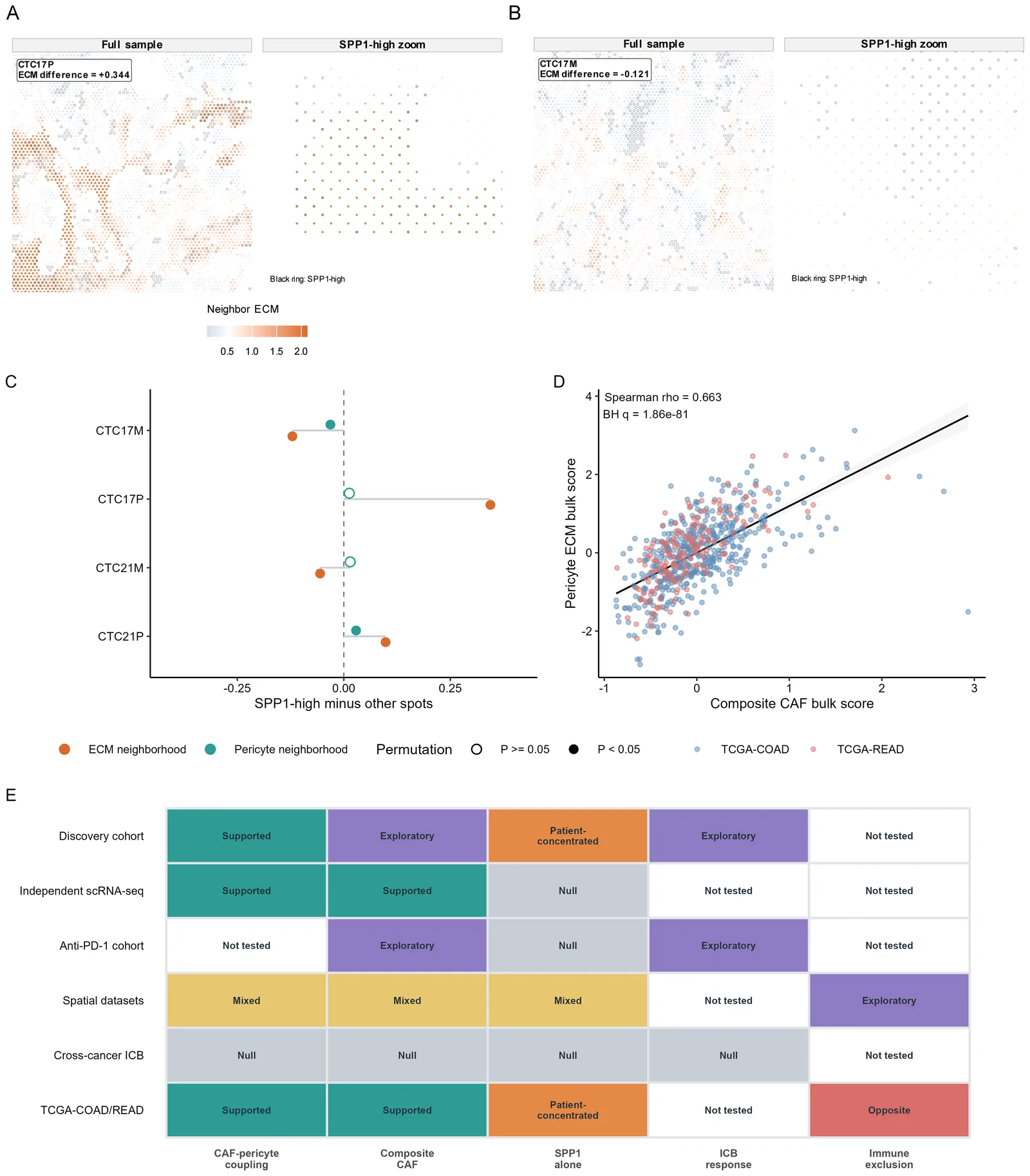
Spatial, cross-cancer immunotherapy, and TCGA boundary analyses**. (A, B)** Representative full-sample and SPP1-high neighborhood views in Visium samples. **(C)** Sample-level ECM and pericyte-neighborhood differences around SPP1-high spots, with within-sample permutation results. **(D)** TCGA-COAD/READ association between composite CAF and pericyte ECM bulk scores. **(E)** Evidence matrix summarizing supported, mixed, null, opposite, patient-concentrated, exploratory, and untested findings. IMvigor210 provides a null cross-cancer boundary test. Spatial observations and Visium spots are not treated as cells, and TCGA is not used for immunotherapy validation.

Across four GSE267401 Visium samples, two primary samples had positive ECM-neighborhood differences around SPP1-high spots, whereas two metastatic samples had negative differences. Pericyte-program differences were small and inconsistent, and within-sample label permutations showed no uniform direction. These data provide no evidence of consistent spatial colocalization between SPP1-high CAF programs and pericytes.

In IMvigor210 urothelial carcinoma, composite CAF, and pericyte ECM signatures showed weak, non-significant associations with non-response. The odds ratios were 1.21 and 1.20 (P = 0.170 and 0.191), with AUC values of 0.561 and 0.554. SPP1 alone had an AUC of 0.524 and did not support a universal cross-cancer immunotherapy biomarker. In TCGA-COAD/READ, the composite CAF score correlated with pericyte ECM (ρ=0.663; FDR = 1.86×10^−81^) and TGF-β/ECM (ρ=0.651; FDR = 1.09×10^−77^). SPP1 also correlated with pericyte ECM (ρ=0.598; FDR = 6.94×10^−63^). However, pericyte ECM and the composite CAF score were positively associated with bulk CD8 cytotoxicity (ρ=0.215 and 0.368), rather than inversely associated (**Figures 6D, E**). Bulk coexpression cannot establish cellular localization, vascular access, or immune exclusion.

## Discussion

This patient-aware analysis identified coordinated fibrovascular transcriptional remodeling in a predominantly CRC immunotherapy cohort. The strongest evidence came from pretreatment matched normal-tumor comparisons within patients, rather than pooled cell-level response contrasts or individual markers. After DecontX correction, tumor pericytes showed higher ECM6, matrix stiffness proxy, and hypoxia/angiogenesis transcriptional program scores, together with increased COL1A1 and FN1. Importantly, the supported ECM6 signal should be interpreted as coordinated basement-membrane transcriptional remodeling rather than uniform upregulation of every constituent gene. The module-level shift remained detectable despite heterogeneous effects among individual components, consistent with the lack of independent significance for COL4A1 and LAMA4. All three prespecified signatures remained significant at the stricter five-pericyte threshold. These findings indicate pericyte transcriptional remodeling, although tissue stiffness, vascular permeability, and immune-cell access were not measured directly.

Technical factors qualify this interpretation. Ambient RNA and doublets can distort mesenchymal profiles in droplet-based data (22–24). Tissue dissociation can also alter stromal recovery and induce stress programs (25, 26). The concordant direction of raw-count, DecontX-corrected, singlet-only, and disjoint-score results argues against a single preprocessing artifact. The absence of a positive association between the CAF:pericyte ratio and paired ECM6 change also weighs against simple stromal loading. However, unfiltered droplets were unavailable, predicted doublets were frequent among pericytes, and several CRC-only singlet estimates were borderline. Residual technical variation therefore remains possible.

The CAF analysis complements the pericyte findings. CAF phenotypes span matrix-producing, inflammatory, and immunomodulatory programs rather than fixed universal categories (10–12). CRC studies also place these programs within multicellular stromal niches (13–15). The 109-cell CAF_C16 split was almost entirely contributed by P02 and nested within a 2,983-cell ECM-remodeling CAF population detected in all 22 patients. After excluding P02, patient-blocked pseudobulk analysis identified matrix and inflammatory programs across 15 patients. There was no evidence that the parent population was enriched in SD, and SPP1 did not pass pseudobulk expression filtering. Thus, the broader ECM-remodeling population, rather than CAF_C16 itself, represents the reproducible CAF signal.

External CRC analyses likewise favored a distributed composite fibroblast program over SPP1 alone or a universal discrete CAF type. In GSE178341, the program mapped to published GREM1-positive and MMP3-positive fibroblast states and correlated with pericyte ECM at the patient level (34). In GSE205506, inflammatory and composite CAF scores were higher in non-pCR samples after fibroblasts were selected using 10 lineage genes independent of the tested scores (2). However, the highest-scoring unsupervised cluster was donor-concentrated and showed no patient-level association with response. The small non-pCR group and unavailable published cell labels limit this cohort to supportive sensitivity evidence.

Communication and spatial analyses further constrained the model. CellChat ranks transcript-compatible ligand–receptor relationships but cannot establish ligand engagement or causal signaling (31–33). The same-patient P02 analysis retained collagen-, laminin-, and FN1-compatible pathways, but its findings cannot be generalized across response groups. The Zenodo spatial resource yielded a negative result (35). Across four Visium samples, spatial directions were mixed, and each spot represented multiple cells (36–38). IMvigor210 did not validate a universal cross-cancer immunotherapy biomarker (39). TCGA showed stromal coexpression, but bulk data cannot resolve cellular localization, vascular access, or immune exclusion (40, 41).

Several design features strengthen the analysis, including matched baseline sampling, reconciled response metadata, patient-level inference, and sensitivity analyses for contamination and doublets. Other strengths include response-blind resolution selection, patient-blocked CAF pseudobulk analysis, a non-overlapping external lineage gate, and explicit reporting of negative results. These features do not remove important limitations. The cohort was modest and enriched for dMMR disease, treatment regimens were heterogeneous, and two patients had duodenal carcinoma. Only two SD patients entered the primary pericyte analysis, and several matched pairs contained few pericytes. Unfiltered droplets and direct mechanical, vascular, and perturbation measurements were unavailable. Larger treated CRC cohorts should use prespecified state mapping, uniform sample processing, direct stromal annotation, spatially resolved cellular assays, and orthogonal functional measurements.

Overall, patient-aware reanalysis links pretreatment pericyte transcriptional remodeling to a cross-patient ECM-remodeling CAF population. The data do not support an SD-associated SPP1-high CAF biomarker or causal CAF-to-pericyte signaling. Instead, they define a fibrovascular remodeling framework that separates reproducible patient-level programs from donor-driven clustering, technical confounding, and context-dependent external evidence (**Figure 7**).

**Figure 7:**
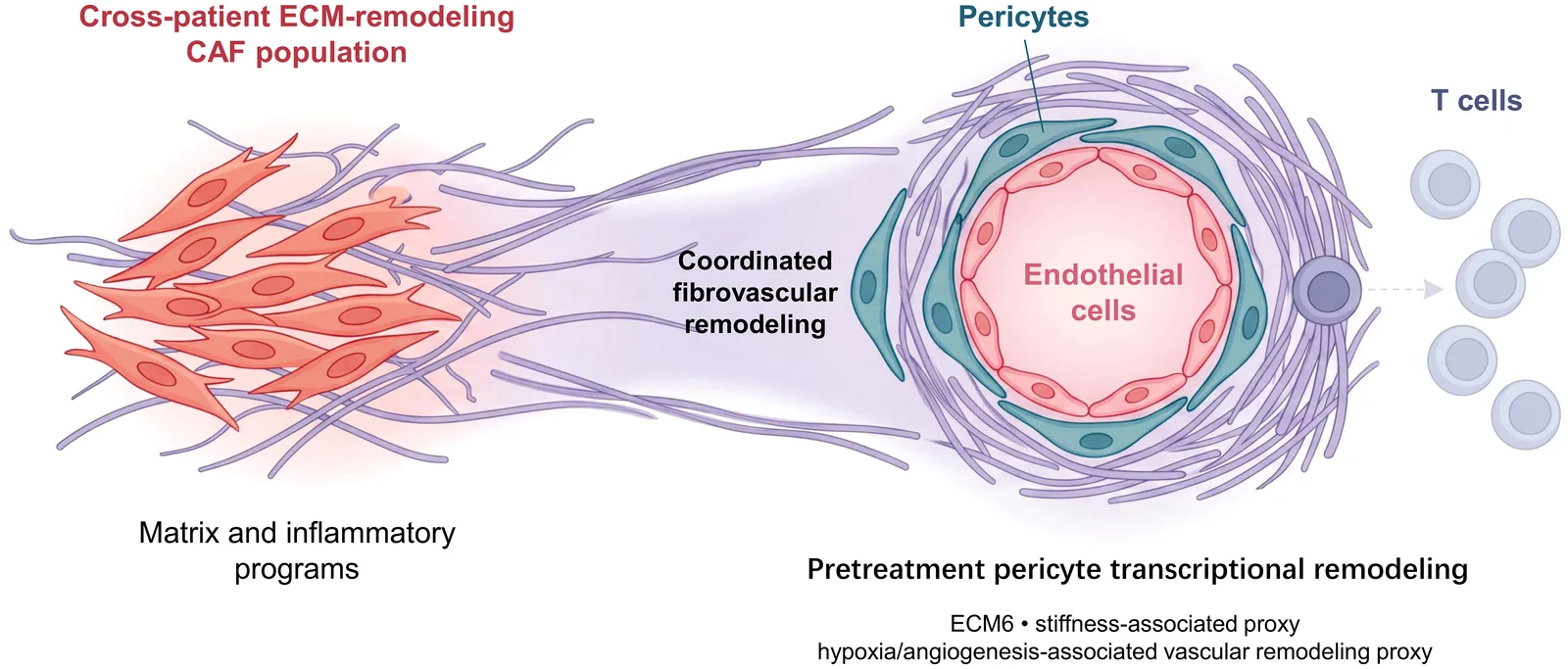
Evidence-bound model of fibrovascular remodeling. Solid elements represent the supported transcriptional observations. Pretreatment tumor pericytes show higher ECM6, matrix stiffness proxy, and hypoxia/angiogenesis transcriptional program scores. A cross-patient ECM-remodeling CAF population also shows matrix and inflammatory programs after P02 exclusion. Dashed elements represent hypotheses. Direct CAF-to-pericyte signaling, effects on vascular mechanics or immune-cell access, and treatment-response consequences remain untested. SPP1 is not a validated state-defining mediator.

## Data Availability

The discovery dataset is publicly available under GEO accession GSE236581. External datasets are available under GSE178341, GSE205506 and GSE267401, Zenodo record 14602110, the IMvigor210 resource and the Genomic Data Commons for TCGA-COAD/READ. **Supplementary Tables S1–S8** report the statistical source data, complete gene-set definitions and sensitivity analyses. All analysis code required to reproduce the revised patient-aware single-cell and downstream analyses is publicly available in the GitHub repository (https://github.com/Giogle-GY/crc-fibrovascular-remodeling-code). A versioned snapshot of the code corresponding to the revised manuscript has also been permanently archived in Zenodo and is available at https://doi.org/10.5281/zenodo.22526263.

## Supplementary material

Supplementary Figure 1 Ambient-RNA, doublet, and baseline pericyte quality-control audit. **(A, B)** Cell-level nUMI and detected-gene distributions in CAFs and pericytes. **(C)** DecontX contamination estimates. **(D)** scDblFinder scores. **(E)** Concordance between raw and DecontX-corrected pericyte ECM6 scores. **(F)** Baseline pericyte counts by patient and tissue. These cell-level panels provide quality-control summaries.

Supplementary Figure 2 Audit of the original P02-dominated CAF_C16 split. **(A)** Original 109-cell split in the CAF UMAP. **(B)** Patient and treatment-stage distribution. **(C)** P02 CAF composition before and after treatment. **(D)** Selected-gene detection within P02 Treatment II; no marker passed correction. **(E, F)** Same-patient, same-stage CellChat results. These panels document why the original split is not used for cohort-level inference.

Supplementary Figure 3 CAF and pericyte abundance normalized to the full quality-controlled cell denominator. **(A)** Matched normal and tumor CAF and pericyte abundances per 1,000 quality-controlled cells across all available tissue stages. **(B)** The same calculation restricted to pretreatment baseline samples. Each line represents one patient; colors denote the official CR, PR and SD response groups. Values are descriptive and are not used in response-associated inferential tests.
